# Zika Virus and Congenital Zika Syndrome: Special Issue Editorial

**DOI:** 10.3390/v18010080

**Published:** 2026-01-07

**Authors:** Marcos Vinicius da Silva Pone, Sheila Moura Pone

**Affiliations:** National Institute of Women, Children and Adolescents Health Fernandes Figueira, Oswaldo Cruz Foundation (IFF-Fiocruz), Rio de Janeiro 22250-020, Brazil; sheila.pone@fiocruz.br

Ten years have passed since the onset of the Zika virus (ZIKV) epidemic in Brazil, which began in 2015 and rapidly evolved into a global public health emergency. Initially recognized for its association with neurological complications in adults, the outbreak soon revealed its most devastating consequence: congenital Zika syndrome (CZS), characterized by severe brain malformations and developmental impairments in newborns [1,2]. This unprecedented event challenged health systems, spurred urgent research, and reshaped our understanding of arboviral infections [3,4]. Today, sustained scientific inquiry remains essential, not only to elucidate the long-term pathophysiological effects of congenital infection but also to ensure comprehensive clinical follow-up and support for affected children and their families. This Special Issue compiles recent evidence on clinical outcomes, diagnostic challenges, immunological mechanisms, and epidemiological trends, aiming to advance scientific understanding, optimize patient care, and inform evidence-based strategies to mitigate the long-term impact of congenital Zika syndrome.

Adam et al. assess ZIKV prevalence and population immunity in Sudan, where epidemiological data are scarce. The study analyzed 198 sera collected in 2012–2013 to detect neutralizing antibodies against ZIKV, dengue virus (DENV), and yellow fever virus (YFV). Neutralization titers were compared among viruses and against the WHO ZIKV antibody standard for ZIKV antibodies. Twenty-six sera neutralized ZIKV, one-third with higher titers than DENV-2 and -3, and two exceeded the WHO standard. Findings suggest occasional ZIKV infections and indicate that during the study period, the population was largely susceptible due to the low percentage of sera with neutralizing antibodies [5].

Piauilino et al. explore whether yellow fever virus (YFV) immunity protects against severe outcomes of ZIKV infection during pregnancy. The study evaluated anti-YFV antibodies and pregnancy outcomes in 172 women with confirmed ZIKV infection during pregnancy in 2016. Outcomes were classified as severe (miscarriage, stillbirth, microcephaly), moderate (low birth weight or preterm delivery), or none. About 89% of participants had YFV immunity, including 100% of severe cases, 84% of moderate, and 89% of none. No significant differences were found for antibody presence (*p* = 0.65) or titers (*p* = 0.6), indicating no protective effect of YFV immunity against severe ZIKV-related outcomes [6].

Emperador et al. evaluate commercial serological assays developed after the 2015 ZIKV epidemic in Brazil. Diagnosing recent infection in pregnant women remains challenging due to antibody cross-reactivity with other flaviviruses (DENV, YFV, WNV). The study assessed the sensitivity and specificity of IgM and IgG tests from multiple manufacturers using panels with known exposures. IgM performance was highly variable; only Inbios 2.0 IgM ELISA showed high sensitivity and specificity. IgG tests were generally sensitive but had variable specificity, with false positives in samples infected by other flaviviruses. Findings confirm challenges in accurate ZIKV testing in flavivirus-endemic regions and underscore the need for standardized reference panels [7].

Martins et al. examine the association between ZIKV infection during pregnancy and congenital CNS abnormalities, including microcephaly. The retrospective cohort analyzed 7870 pregnant women in Rio de Janeiro, Brazil, 2269 infected and 5601 non-infected, along with their fetuses and newborns. Using state databases and propensity score modeling, 49 cases of microcephaly or CNS anomalies were identified among infected women (2.16%) versus 44 (0.78%) in non-infected. The odds ratio was 2.46 overall, with highest risk in the first (OR 4.29) and second trimesters (OR 5.29). Frequent findings included intracranial calcifications, ventriculomegaly, posterior fossa malformations, reduced brain volume, corpus callosum malformations, cortex dysplasia, lissencephaly, and pachygyria. Ophthalmologic and auditory anomalies occurred in 55.5% and 33.3% of cases. Given the absence of specific treatment, clinical care should prioritize monitoring neurological, motor, auditory, and visual disorders in all children with in utero ZIKV exposure, especially during the first and second trimesters of pregnancy [8].

Martins et al. describe neurological, visual, and auditory outcomes in children whose mothers had confirmed ZIKV infection during pregnancy, most without microcephaly. The observational, longitudinal, prospective study in Rio de Janeiro (2015–2017) followed exposed children for up to 30 months. Among 2882 pregnant women, 116 had suspected infection and 33 were laboratory confirmed. Only one child had congenital microcephaly; however, neurodevelopmental delay occurred in 36.4% of the evaluated children, radiological abnormalities in 29.1%, auditory issues in 8.3%, and ophthalmological problems in 10%. Findings highlight that ZIKV exposure may cause significant impairments even without congenital microcephaly, reinforcing the need for long-term monitoring and early intervention [9].

Ju et al. focus on identifying diagnostic characteristics of CZS using advanced imaging. CZS, caused by ZIKV infection during pregnancy, often leads to microcephaly due to severe brain volume reduction and impaired growth. While screening typically relies on head circumference measurements, 3D head features analyses have been limited. The study compared 3D head images of 35 ZIKV-positive cases (mean age 16.8 months) and 35 controls (14.4 months). Significant differences were found in centroid size, head circumference (HC), head height (HH), and chin height (CH). Ratios such as CH/TFH (total facial height) and HH/HC also differed; an HH/HC ratio of 0.49 achieved sensitivity of 0.86 and specificity of 0.74 for diagnosing CZS, outperforming HC alone. HH/HC shows promise as a more sensitive diagnostic tool for early CZS detection [10].

Rodrigues et al. assess clinical and acoustic swallowing alterations in children exposed to ZIKV during pregnancy, focusing on oropharyngeal dysphagia (OD), common in CZS. The case series included 22 children from Amazonas, Brazil (6 with microcephaly, 16 without; mean age 35 months). All microcephalic children showed oral motricity changes, mainly in lips and cheeks, plus vocal and palatal alterations; half had cervical auscultation changes during breast milk swallowing. Non-microcephalic children most often had lip motricity issues without auscultation changes. For liquid and pasty foods, frequent problems included incomplete labial closure, prolonged oral transit time, spoon capture inadequacy, and anterior labial leakage. These findings underscore the need for comprehensive swallowing evaluations in all ZIKV-exposed children [11].

Da Silva et al. explore the relationship between sleep patterns and language development in children with CZS. The study included a cross-sectional analysis of 135 children (0–48 months) using BISQ for sleep and the Early Language Milestone Scale for language, plus Bayley Scales for motor, cognitive, and social abilities. Additionally, a longitudinal cohort of 16 children was followed for four years. Sleep disturbances and language deficits were frequent. In 0–12 months, late bedtime and frequent awakenings correlated with poorer auditory expressive skills; at 13–24 months, nighttime awakenings remained associated with expressive deficits; and at 25–36 months, decreased auditory receptive skills were linked to longer sleep onset and reduced nighttime sleep. Findings suggest ZIKV-related brain alterations affect both sleep and language development, and sleep disturbances may mediate the pathway between CZS and delayed language acquisition, as all three analyzed language skills correlated with sleep parameters [12].

Bezerra et al. investigate the peripheral dysregulation of immune mediators in children with CZS-related microcephaly. Gene expression analysis using qPCR in whole blood samples revealed increased IFNγ and IL-13 transcripts in affected children versus controls, while serum assays showed decreased CCL2 and CXCL8. Allergy prevalence was high, corroborated by elevated IgE levels measured by the Proquantum Immunoassay. These findings indicate persistent systemic inflammation and impaired leukocyte migration caused by low CCL2 and CXCL8 production, alongside high IgE levels associated with allergy prevalence. Dysregulation of inflammatory genes and chemokines underscores the need for research on long-term consequences to guide developing targeted therapies and tailored vaccination strategies [13].

This Special Issue underscores the profound and lasting impact of ZIKV infection, particularly congenital Zika syndrome, on global health. Despite significant advances in understanding clinical outcomes, diagnostic limitations, immunological mechanisms, and neurodevelopmental consequences, critical knowledge gaps remain. Long-term follow-up of affected children, refinement of diagnostic tools, and exploration of therapeutic and preventive strategies are essential to mitigate the enduring burden of CZS. Continued multidisciplinary research is imperative—not only to address unresolved questions but also to strengthen preparedness for future arboviral threats.
